# A Clinical Perspective on the Dietary Therapies for Pediatric Eosinophilic Esophagitis: The Gap Between Research and Daily Practice

**DOI:** 10.3389/fimmu.2021.677859

**Published:** 2021-05-19

**Authors:** Liselot De Vlieger, Lieselot Smolders, Lisa Nuyttens, Sophie Verelst, Christine Breynaert, Tim Vanuytsel, Ilse Hoffman, Dominique MA Bullens

**Affiliations:** ^1^ Allergy and Immunology Research Group, Department of Microbiology, Immunology and Transplantation, KU Leuven, Leuven, Belgium; ^2^ Department of General Internal Medicine, UZ Leuven, Leuven, Belgium; ^3^ Clinical Division of Pediatrics, UZ Leuven, Leuven, Belgium; ^4^ Gastroenterology and Hepatology, UZ Leuven, Leuven, Belgium; ^5^ Pediatric Gastroenterology, Hepatology and Nutrition, UZ Leuven, Leuven, Belgium

**Keywords:** eosinophilic esophagitis, pediatric, dietary therapy, elemental diet, elimination diet, allergy-test directed diet, reintroduction, child

## Abstract

Pediatric eosinophilic esophagitis (ped-EoE) is an immune-mediated pathology affecting 34 per 100.000 children. It is characterized by an esophageal inflammation caused by an immune response towards food antigens that come into contact with the esophageal lining. Depending on the age of the child, symptoms can vary from abdominal pain, vomiting and failure to thrive to dysphagia and food impaction. The diagnosis of this chronic disease is based on the symptoms of esophageal dysfunction combined with an infiltration of more than 15 eosinophils per high-power field and the exclusion of secondary causes. The treatment modalities include the 3Ds: Drugs, allergen avoidance by Diet and/or esophageal Dilation. In this review we focused on the efficacy of dietary approaches in ped-EoE, which currently include the elemental diet (amino acid-based diet), the empiric elimination diet and the allergy test-directed elimination diet. Although several reviews have summarized these dietary approaches, a lack of consistency between and within the elimination diets hampers its clinical use and differences in subsequent reintroduction phases present a barrier for dietary advice in daily clinical practice. We therefore conducted an analysis driven from a clinician’s perspective on these dietary therapies in the management of ped-EoE, whereby we examined whether these variations within dietary approaches, yet considered to be similar, could result in significant differences in dietary counseling.

## Introduction

Pediatric eosinophilic esophagitis (EoE) is an immune-mediated disease of the esophagus with an overall prevalence of 34 cases per 100.000 children (1). EoE is clinically characterized by esophageal dysfunction and histologically by an eosinophilic infiltration of more than 15 eosinophils (eos) per high-power field (HPF) in biopsies taken from the proximal and/or distal esophagus (2). In infants and toddlers symptoms can range from food refusal, slow eating, abdominal pain, vomiting and less commonly failure to thrive while school-aged children and adolescents rather suffer from dysphagia, food impaction and chocking (2, 3). This inflammatory disease has a multifactorial etiology. Both immunogenetic and environmental factors might contribute to the T helper (Th) 2-driven allergic immune response towards primarily food antigens, specific for each patient (2). Hereby, recent studies hypothesize that this food-antigen mediated disease could be independent of IgE and rather driven by an IgG_4_-mediated response (2, 4).

The treatment of EoE revolves around the 3Ds: Drugs, allergen avoidance by Diet and/or esophageal Dilation. The advantage of a dietary therapy is that it can target the cause of the disease, while drugs and dilation therapy mainly treat the symptoms of the disease. Currently, there are three dietary therapies for EoE: the elemental diet also known as the amino acid-based elemental diet (ELED), the empiric elimination diet and the allergy test-directed elimination diet. In the amino-acid based ELED all potential food triggers are eliminated, since this diet exclusively consists of hypoallergenic amino acid-based formulas (5). This contrasts to the “empiric elimination diet”, where only one or more subset(s) of food triggers that are most commonly associated with food allergy and/or esophageal eosinophilia are eliminated (5). Thirdly, the allergy test-directed elimination diet, eliminates specific food triggers based on the results of allergy tests (5). Until now, several meta-analytic reviews have been performed on the efficacy of these dietary therapies in EoE (3, 5–7). However, when comparing the studies that formed the base for these reviews we found that the prescribed diets in these trials vary substantially, which makes dietary advice still a remaining challenge in daily clinical practice.

The aim of this review was to conduct an analysis driven from a clinical perspective on the different dietary therapies in the management of pediatric EoE patients evaluated by an endoscopy. We thereby searched for potential differences between and within the published elimination diets and subsequent reintroduction phase and evaluated whether these variations could actually lead to important differences in dietary counseling.

## Methods

### Study Selection

**Figure 1:**
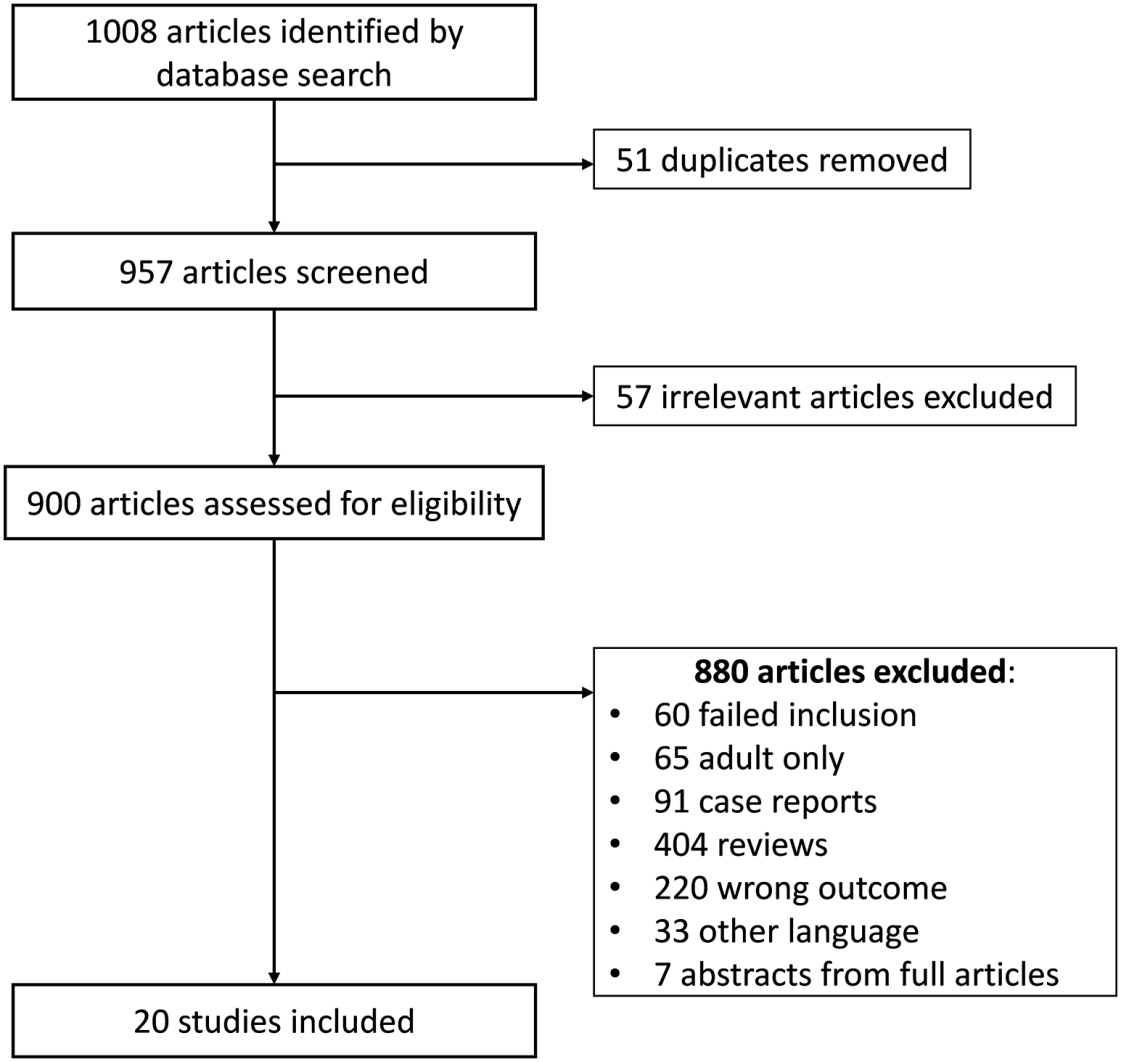
Flow chart of study selection.

Four bibliographic databases (PubMed, Embase, Scopus & Web of Science) were searched from January 1995 till December 2020. The following meSH terms were used to identify articles concerning the dietary therapy of EoE during childhood (0-18 years): eosinophilic esophagitis and therapy and diet and children (**Figure 1**).

English written randomized controlled trials and observational prospective and retrospective studies were included if histological remission was considered as an endpoint after dietary therapy. This required an esophagogastroduodenoscopy (EGD) at the beginning and at the end of the study, whereby the diagnosis of EoE was confirmed by an esophageal biopsy showing > 15 eos/HPF. The histological remission after the dietary treatment was defined as the actual accepted remission state at the time of publication of the study: initially considered as a reduction of the number of eosinophils in the esophageal biopsies and later on when the peak of the eosinophil count in the biopsy was below 15, 10 or 5 eos/HPF (8). The following dietary interventions were analyzed: elemental diet (ELED), allergy test-directed elimination diet, empirical 6-food elimination diet (6-FED), 4-food elimination diet (4-FED), two-food elimination diet (2-FED) and one-food elimination diet (1-FED). Studies that combined dietary therapy with medication, or compared diet and medication were not excluded. However, studies involving only adults, studies without an endoscopic evaluation at the beginning and at the end of the dietary intervention for each patient and case reports were excluded. When there was any indication that patients might be involved in multiple studies, we only included the most recent study with the largest number of patients of this research group. This rule also applied to duplicated information (e.g. abstracts prior to a full paper).

### Classification Based on Self-Defined Levels of Proof

The incorporated studies were not classified according to the classical CEBM evidence levels, since we wanted to focus on both study design and the quality in which the dietary restrictions were monitored. We have therefore established an author-based standard to approach the evaluation of the quality of the included studies, considering the fact that the intervention was a diet and that the quality of the follow-up and/or dietary counseling may have influenced the results. Hereby, studies were classified according to four self-defined levels of proof ranging from A (highest level) to D (lowest level) (see **Table 1**). We required self-defined levels of proof as most trials did not perform any randomization or assigned subjects to a specific group solely based on allergy testing, prior choice of patient/physician or age. As a result, most clinicians were not blinded for the dietary treatment or only the pathologist performing biopsies was unaware of the clinical information, whereas the treating physician remained unblinded. Finally, at the time we performed this review no results of placebo-controlled dietary trials concerning pediatric EoE were available.

**Table 1:** Level of proof of the pediatric EoE studies.

Level of proof	Explanation
A	Prospective interventional and/or observational studies.
B	Retrospective interventional and/or observational studies in which the patients were strictly monitored and had extensive counseling with interim follow-up by a dietician or study nurse.
C	Retrospective interventional and/or observational studies in which the patients received instructions regarding the dietary therapy at the beginning of the study, but data on interim follow-up were lacking.
D	Abstracts, notes of conference proceedings, incomplete reports and letters to the editor.

### Analysis

We used the statistical software program OriginPRO 2021, and presented the results for each dietary therapy by 3D histograms combining the number of included patients and the level of proof. If the number of children in the intervention group was not specified, we anticipated that it was unlikely that this number was higher than in most other studies with the same diet. We therefore approached the unknown by calculating the average number of patients participating in the included pediatric EoE studies analyzing the efficacy of the same diet by dividing the total number of participants described in those other studies by the number of studies performed with the same diet. The remission rates of the dietary therapies were calculated as weighted arithmetic means thereby considering the number of patients in a study as well as the histological effect of the diet.

## Results

Based on the search strategy 20 studies were found, of which 16 original full articles (9–24) and four abstracts (25–28). Nine studies assessed the effect of one dietary therapy (10, 14, 15, 19, 20, 22, 25, 27, 28), while eleven studies evaluated the efficacy of several diets (9, 11–13, 16–18, 21, 23, 24, 26). Concerning the effect of medical therapy, one study compared the effect of cow’s milk elimination and topical corticosteroids (tCS) (22) while a second study assessed the efficacy of a combined and alternating therapy with tCS and 2-FED (14). A total of 1220 children were included in this analysis, with study size per dietary therapy ranging from 3 to 164 patients.

### Amino Acid-Based or Elemental Diet (ELED)

**Figure 2:**
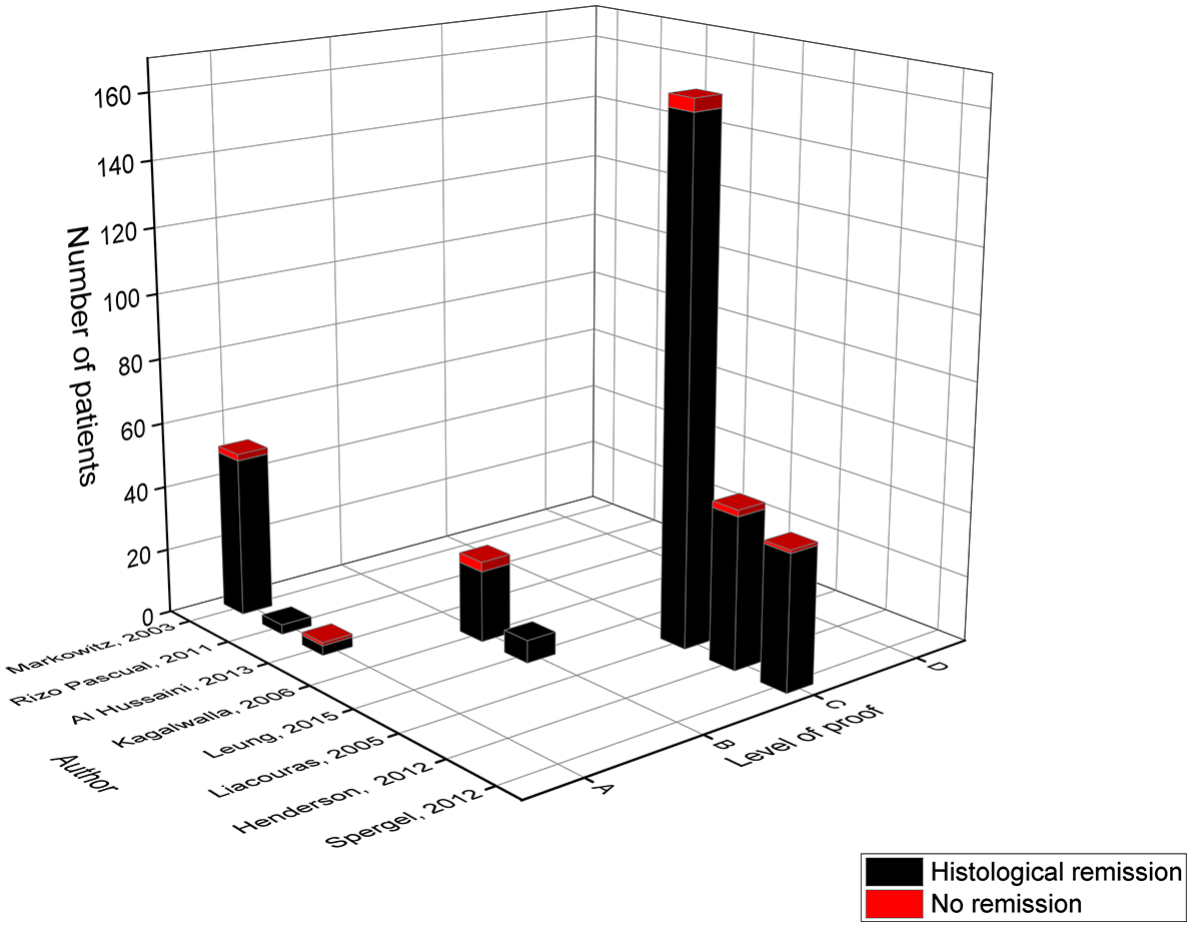
Level of proof of studies on the elemental diet.

The efficacy of the ELED for the treatment of EoE was first described in 1995 by Kelly et al. (29). This diet exclusively consists of amino acids, and was assessed in eight pediatric studies with a total of 352 patients and a mean histological remission in 96% of the children (**Table 2** and **Figure 2**). The duration of the ELED ranged from four to eight weeks in most of the studies, after which a repeat endoscopy was performed to evaluate the histological remission.

**Table 2:** Overview of studies on the elemental diet.

Level	Author, publication year	Mean age (years)	n	Period	Duration of diet	PPI	Histological response(eos/HPF)	Remission rate
A	Markowitz, 2003	8,3 (3–16)	51	1997-2000	4 weeks	Yes, NR	Reduction	96%
A	Rizo Pascual, 2011	9 (2,8–14,5)	3	2001-2009	8 weeks	NA	<10	100%
A	Al Hussaini, 2013	1,5 (1–2)	4	2009-2012	8 weeks	NA	≤ 5	75%
B	Kagalwalla, 2006	6,4	25	2001-2003	6 weeks	NR	≤ 10	88%
B	Leung, 2015	13 (8–18)	7	2007-2013	8 weeks	Yes, NR	< 10	100%
C	Liacouras, 2005	8,1	164	1994-2004	4-5 weeks	NR	Reduction	97%
C	Henderson, 2012	5,6 (0,9–19,7)	49	1999-2011	Mean 4.5 months	NR	< 15	96%
C	Spergel, 2012	6,4 (1–18)	***43***	2000-2011	NA	NR	≤ 15	98%

NA, not applicable; PPI, proton pump inhibitors; NR, non-responder; R, responder; eos/HPF, eosinophils per high-power field. Bolded value: the number of children in the intervention group was not specified, we therefore approached the unknown as stated in the method section “Analysis”.

The main effect in level A came from the study of Markowitz in 2003 with a histological remission of 96%, as the more recently published studies of Rizo Pascual and Al Hussaini only included three and four patients respectively (9, 10, 12). However, the study of Markowitz could have overestimated the effect of this diet, since their primary outcome was a reduction of eosinophils in the esophageal biopsies (10) whereas the more recent studies used an absolute eosinophil count below a specific threshold (9, 12). Moreover, children were allowed to consume one fruit (*grape* or *apple*) and its corresponding *pure juice* (10). The small study of Al Hussaini had the lowest effect size in comparison to the six other studies, in which only three of the four children achieved histological remission, but the authors attributed it to non-adherence to the ELED in one child (12). The study of Kagalwalla in 2006 (level B) compared two cohorts of children from two different time periods, one was treated with the ELED while the other followed the 6-FED (18). They achieved 88% remission with the ELED, and achieved histological improvement in four of the five children who failed the 6-FED diet. In the study of Leung in 2015 (level B) all seven children who followed the ELED achieved remission (16). This study also evaluated the histological outcome of the ELED as a rescue therapy after the initial treatment with medication or a diet failed, resulting in a success rate of 100% (16).

The majority of the children involved in the analysis of the ELED came from studies categorized in level C. These studies were classified according to level C since they are retrospective without data on intermediate follow-up to monitor dietary compliance. In contrast to level A and B studies, these studies included children over a time-period of at least ten years. Overall, the study of Liacouras was the largest study achieving remission in 97% of the children (13). Again, it should be noted that these authors studied the reduction in eosinophil count instead of an absolute eosinophil count. This study also allowed *white grapes* or *apples* and their corresponding *juice* (10, 13). In addition, only the study of Liacouras mentioned performing allergy tests to apples and grapes before introducing one fruit into the daily diet (13). In 2012, the studies from Henderson and Spergel achieved respectively 96% and 98% of remission (21, 23). Hereby, the study of Spergel assessed the ELED in only 5% of its study population, while 165 children received 50% of their calorie-intake through the ELED. Depending on the results of the skin prick test and atopy patch test limited foods (not defined) were added to their daily diet (21). However, these results were not included in the comparative analysis as this was not a pre-specified diet.

#### The ELED in Daily Practice

In clinical practice the use of an ELED in older children is affected by the disadvantages of being exclusively fed for a long-term period on amino acid formula. The poor palatability of the formula has a major impact on the implementation and as a result most of the studies administered the formula by a nasogastric tube rather than orally. Secondly, removing all solid food from the youngest children during their first years of life may have a negative impact on both feeding and speech skills while also increasing the risk of developing a food allergy (7). To compensate for these hurdles two studies allowed their participants to consume *grapes* or *apples* and their corresponding *juice* (10, 13). This raises the question as to why these two types of fruit were specifically chosen? Indication of eosinophilic gastrointestinal disease caused by apple consumption, in a child with birch pollen allergy due to PR10 sensitization, exists (30). It is possible that children with birch pollen allergy might react to apple and/or grapes with eosinophilic esophageal inflammation too. Would it be useful to choose one or two fruits allowed in the diet for all patients separately? However, even if we could overcome these hurdles by using one/two fruits in addition to the ELED, we still need to identify the culprit food after the ELED in which a large number of endoscopies have to be performed. Finally, the ELED is very expensive due to the suspension itself as well as the medical costs linked to the frequently opted tube feeding.

In conclusion, the ELED is highly effective in the management of pediatric EoE, but should rather be used in formula fed infants or as a final rescue therapy for older children who did not experience histological remission on other dietary therapies (7, 31, 32).

### Allergy Test-Directed Elimination Diet

**Figure 3:**
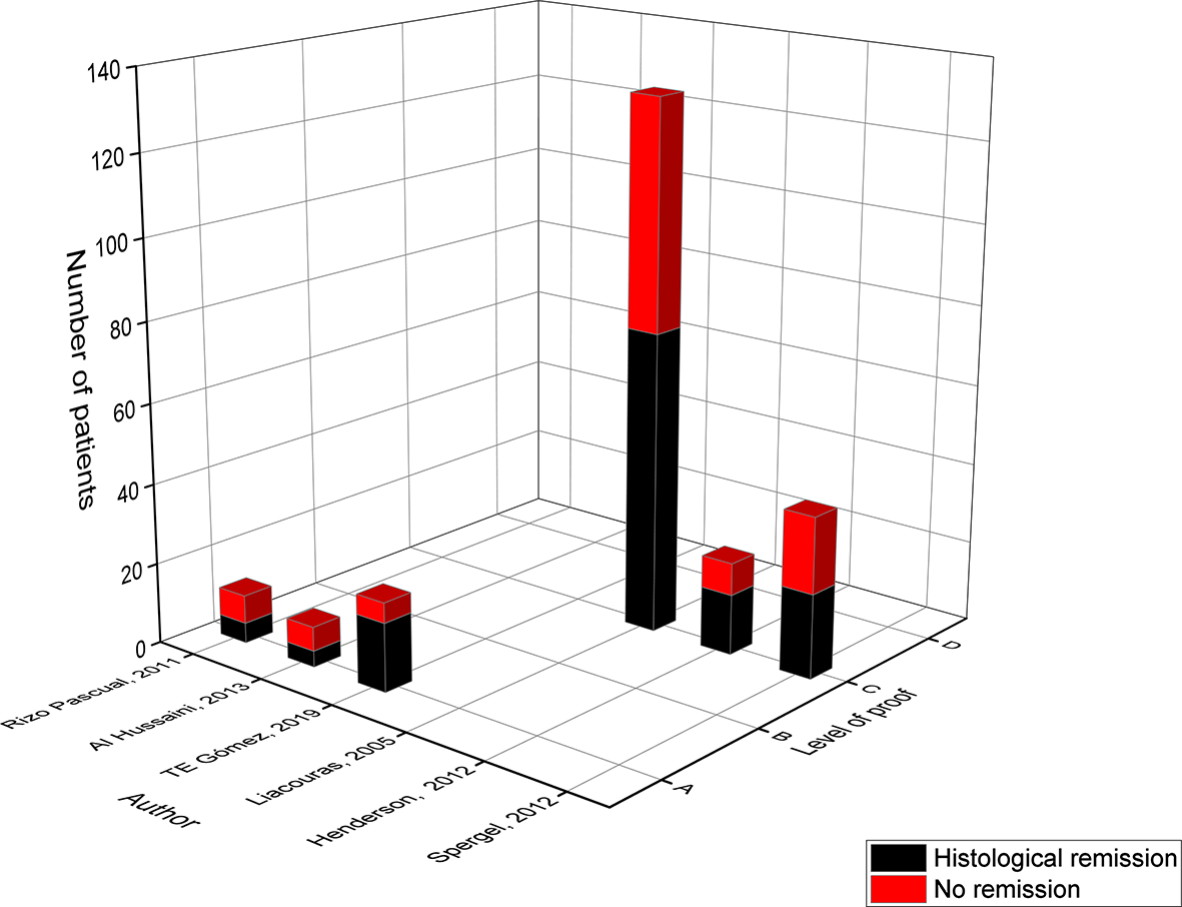
Level of proof of studies on the allergy test-directed elimination diet.

Jonathan M. Spergel was the first who published the effect of an elimination diet in a pediatric population, based on the results of the skin prick test (SPT), atopy patch test (APT) and serum specific IgE (sIgE) levels (33). Since then, six pediatric studies implemented a diet based on positive allergy tests with a total of 239 children and a mean histological remission of **58%** (**Table 3** and **Figure 3**) (9, 11–13, 21, 23). Five studies used the SPT as allergy test (9, 12, 13, 21, 23), four studies the APT (9, 13, 21, 23) while only three studies measured the sIgE levels (9, 11, 12). Most of these studies achieved a low histological remission rate between 40% and 50%, in contrast to the level A study of TE Gómez in 2019 and level C study of Henderson in 2012 who were able to reach remission in respectively 77% and 65% of the children (11, 23). The level A study of TE Gómez was the first to exclusively evaluate the sIgE-based elimination diet in a pediatric population (11). Food products were eliminated if the sIgE levels were ≥ 0.1 kU/L for cow’s milk, wheat, egg, lentils, peanuts and hake/shrimp. This study differs from the level A studies of Rizo Pascual in 2011 and Al Hussaini in 2013, who on top of the skin tests also measured the sIgE levels and used a higher cut-off value of 0.35 kU/L (9, 12). The study of Al Hussaini eliminated foods that were identified by a positive SPT to cow’s milk, egg white, soy/legumes, wheat, peanut, fish, shrimp, chicken and/or positive sIgE levels to cow’s milk, egg, soy, wheat, fish and peanut (12). This differs from the study of Rizo Pascual where they only measured the sIgE levels of allergens with a positive result on the SPT and/or APT to cow’s milk, egg, chicken, beef, fish, soy/legumes and nuts (9). These two smaller studies induced remission in approximately 40% of the children while the study of TE Gómez achieved a high remission rate of 77% (9, 11, 12).

**Table 3:** Overview of studies on the allergy test-directed elimination diet.

Level	Author, publication year	Mean age (years)	n	Period	Duration of diet	PPI	Allergen evaluation	Histological response(eos/HPF)	Remission rate
A	Rizo Pascual, 2011	9 (2,8–14,5)	12	2001-2009	8 weeks	NA	SPT, APT & Immunocap	< 10	42%
A	Al Hussaini, 2013	6,8 (4–11)	10	2009-2012	8 weeks	NA	SPT & Immunocap	< 15	40%
A	TE Gómez, 2019	10,4 (5–15)	22	2011-2016	6 weeks	NA	Immunocap	< 15	77%
C	Liacouras, 2005	10,4	132	1994-2004	4-5 weeks	NR	SPT & APT	Reduction	57%
C	Henderson, 2012	5,2 (0,9–15,0)	23	1999-2011	Mean 3,9 months	NR	SPT & APT	< 15	65%
C	Spergel, 2012	6,4 (1–18)	***40***	2000-2011	NA	NR	SPT & APT	≤ 15	53%

NA, not applicable; PPI, proton pump inhibitors; NR, non-responder; R, responder; eos/HPF, eosinophils per high-power field; SPT, skin prick test; APT, atopy patch test. Bolded value: the number of children in the intervention group was not specified, we therefore approached the unknown as stated in the method section “Analysis”.

In a study by Leung 54 children were put on a multiple food elimination diet (MFED) based on both clinical (history of IgE food allergies, allergy tests…) and social (preference, social environment…) factors, reaching a remission rate of 63% (16). However, the elimination of a food group did not solely depend on the results of the SPT, APT and/or sIgE levels. Consequently, these results were not used in the comparative analysis and hence not represented in the figures (16). Overall, the study of Liacouras in 2005 (level C) was the largest study with 132 children following an elimination diet based on the results of the SPT to 12 allergens: cow’s milk, egg, soy, wheat, beef, rye, peanut, chicken, corn, peas, potato, rice and/or APT to 10 allergens: soy infant formula, skim milk powder, dried egg white, wheat, oats, barley, rye four, rice flour, corn meal and dehydrated potatoes (13). After four to five weeks, 57% of the children achieved full remission defined by a reduction of eosinophils in the biopsy. However, similar to other studies the authors could have overestimated the success rate of this diet since no absolute eosinophil count was used to assess histological remission (13). In 2012, the two level C studies from Henderson and Spergel combined the results of SPTs and APTs to elicit foods and achieved respectively 65% and 53% of remission (21, 23). In the study of Henderson children were exposed to no less than 62 foods during the SPT (23). This differs from the five other studies, where considerably fewer foods were tested.

#### The Allergy Test-Directed Elimination Diet in Daily Practice

In 2017 the evidence-based statements and recommendations on EoE strongly advised against the allergy-test-directed elimination diet as a treatment option for pediatric EoE, due to the variable histological resolution rates (32). However, several factors could have caused the variable remission rates of this elimination diet which varied from 40% to 77% in the pediatric EoE studies. First of all, the varying panel of food allergens chosen to perform the SPT, APT and/or measure sIgE levels. Indeed, most of the studies focused their search on allergens known to be relevant for EoE such as; cow’s milk, egg, wheat and soy/legumes. Additional allergens that were regularly tested included meats (chicken, beef and turkey), fish, grains (rice, corn, barley and oat), nuts and to a lesser extent potatoes, fruits and vegetables. Secondly, the selected allergy tests (SPT, APT and/or Immunocap) and threshold for considering a positive result which varied substantially amongst the different studies. Thirdly, the geographical area studied, which is now focused in the United States and Spain for the pediatric EoE studies, since this partially determines the food pattern of the participants.

Ultimately, the predictive values of the SPT and APT for the most prevalent food triggers of pediatric EoE are weak. As a result, further research is needed on the variable elements listed above before we can decide whether or not the allergen-directed diet is a good or poor dietary therapy, especially in children with EoE.

### 6-Food Elimination Diet (6-FED)

**Figure 4:**
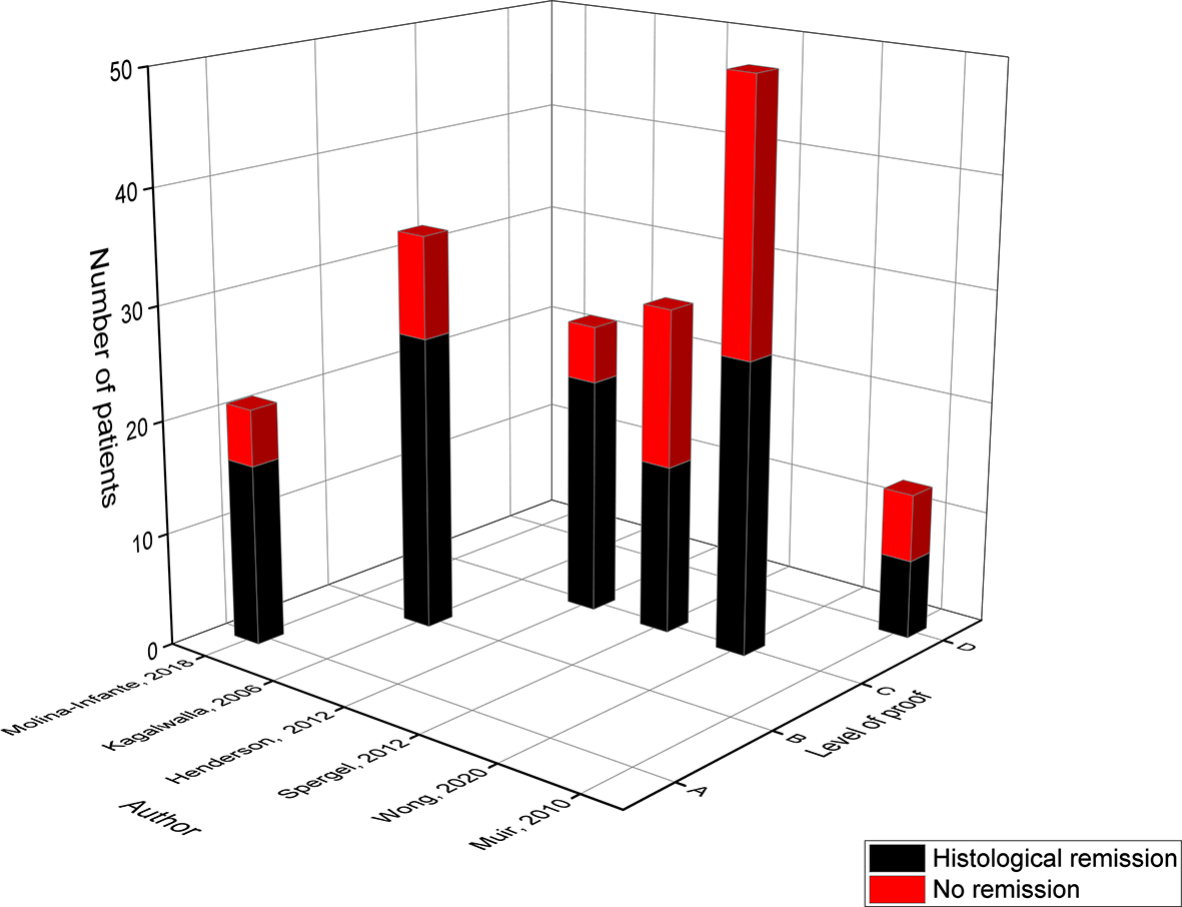
Level of proof of studies on the 6-food elimination diet.

In 2006, Amir Kagalwalla developed the 6-food elimination diet, in which the six most common foods associated with food allergy and mucosal damage in pediatric EoE are removed: cow’s milk, wheat, egg, soy, peanuts/tree nuts and fish/seafood (18). In total, this diet was evaluated in six pediatric studies including 174 patients and resulted in a mean histological remission of 64% (**Table 4** and **Figure 4**) (17, 18, 21, 23, 24, 27).

**Table 4:** Overview of studies on the 6-food elimination diet.

Level	Author, publication year	Mean age (years)	n	Period	Duration of diet	PPI	Diet (Exclusion of)	Histological response(eos/HPF)	Remission rate
A	Molina-Infante, 2018	11 (5–13)	21	2014-2016	6 weeks	Yes, NR	All dairy products, gluten-containing grains, egg, legumes, all nuts, fish & seafood	< 15	76%
B	Kagalwalla, 2006	6,2	35	2003-2005	6 weeks	NR	Cow’s milk, wheat, soy, egg, peanut, tree nuts, fish & seafood	≤ 10	74%
C	Henderson, 2012	6,6 (2,2–20,8)	26	1999-2011	Mean 4,4 months	NA	Cow’s milk, wheat, soy, egg, peanut, tree nuts, fish & seafood	< 15	81%
C	Spergel, 2012	6,4 (1–18)	***29***	2000-2011	NA	NR	Cow’s milk, wheat, soy, egg, peanut, tree nuts, fish & seafood	≤ 15	53%
C	Wong, 2020	7,6 (1–20)	50	NA	Mean 80,3 days	Yes	Dairy, wheat, egg, soy, nuts & seafood	< 15	52%
D	Muir, 2010	9 (1–15)	13	NA	6 weeks	NA	Cow’s milk, wheat, soy, egg, peanut, tree nuts, seafood & corn	>50% reduction	53%

NA, not applicable; PPI, proton pump inhibitors; NR, non-responder; R, responder; eos/HPF, eosinophils per high-power field. Bolded value: the number of children in the intervention group was not specified, we therefore approached the unknown as stated in the method section “Analysis”.

The retrospective study of Kagalwalla in 2006 (level B) was the first that assessed the 6-FED for 6 weeks, reaching a remission rate of 74% (18). In 2012, Henderson (level C) modified the 6-FED and combined the elimination of the six most prevalent food triggers of EoE with the avoidance of food for which the SPT and APT were positive. In this study, 11 children followed the classic 6-FED whereas 15 children were put on the modified 6-FED reaching a high remission rate of 82% and 80% respectively (23). Hereby, the extended therapy duration of 4.4 months could have contributed to the high success rate of this study (23).

In contrast, the study of Spergel in 2012 (level C) and Muir in 2010 (level D) achieved a low histological remission rate of both 53% (21, 27). When the mean eosinophil count was lowered to 10 eos/HPF or less, the prospective study of Muir reached an even lower remission rate of 38% (27). In this study dietary compliance was rated as poor which probably resulted in the low remission rates. In addition, one has to mention that this study modified the 6-FED allowing the consumption of *fish*, while adding the elimination of corn to a diet free of seafood, cow’s milk, wheat, egg, soy and peanut/tree nuts. This low success rate was reproduced in the recent single-center study of Wong (level C), in which only 52% achieved histological remission (24). This study also adjusted the typical composition of the 6-FED and advised to eliminate dairy, wheat, egg, soy, nuts and seafood but apparently did not recommend the avoidance of *peanuts* and *fish* (24). However, higher remission rates were established when the response rate to the 6-FED was analyzed after 10 weeks (68.8%) in comparison to 10-12 weeks (50%) or more than 12 weeks (40%) of treatment (24). Furthermore, children older than 12 years seemed to reach higher remission rates of 72.7% in comparison to children younger than 6 years (36.4%) or between 6-12 years (58.8%) of age (24). Although these effects were not significant, this is the first study showing that the success rate of a diet could be influenced by a shorter treatment duration and an older age group of participants (24).

In 2018 the prospective study of Molina-Infante (level A) evaluated the 6-FED which besides cow’s milk, wheat, egg, tree nuts, fish, seafood and legumes (peanuts, soy, lentils, chickpeas, peas & beans) also avoided the potential cross-allergens of milk (goat’s milk & sheep’s milk) and wheat (gluten-containing cereals barley, rye & oat) (17). The study proposed a step-up dietary therapy for the management of EoE starting with a 2-FED (all dairy products & gluten-containing cereals), since numerous studies identified cow’s milk and wheat/gluten as the most common food triggers. If the children were not in remission after this 2-FED they evolved to a 4-FED (2-FED + egg & soy/legumes) and ultimately a 6-FED (4-FED + all kind of nuts & fish/seafood). In that way the amount of endoscopic procedures was significantly reduced. The remission rates of the 2-FED, 4-FED and 6-FED were 43%, 60% and 76% respectively among the children who completed the study. However, the high success rate of the 6-FED could have been an overestimation since the study focused on children who previously failed the 2- or 4-FED, which resulted in a study dropout rate of 28%.

#### The 6-FED Diet in Daily Practice

It remains difficult to compare data on the 6-FED due to the variability in the food groups that were being eliminated, alongside with the variation in the treatment duration ranging from 6 to 22 weeks. This lack of uniformity in the treatment courses could have led to the variable success rate of the 6-FED which ranged from 52% to 81%. In addition, several studies mentioned poor dietary compliance and high dropout rates when children were put on a 6-FED. However, in comparison to the ELED only a limited number of endoscopies have to be performed in search for the culprit food.

In conclusion, several studies adjusted the composition of the 6-FED which makes it difficult to compare the effectiveness of this diet between trials. Therefore, we first need to assess whether adding foods such as goat’s milk, sheep’s milk, corn, legumes and/or gluten to the 6-FED has a beneficial effect on the histological remission rates. Hereby it is necessary that studies define which type of legumes they eliminate, since only one study mentioned this (17). Secondly, additional studies should clarify whether leaving out fish and peanuts from the list of eliminated foods is changing the efficacy of the 6-FED as well.

### 4-Food Elimination Diet (4-FED)

**Figure 5:**
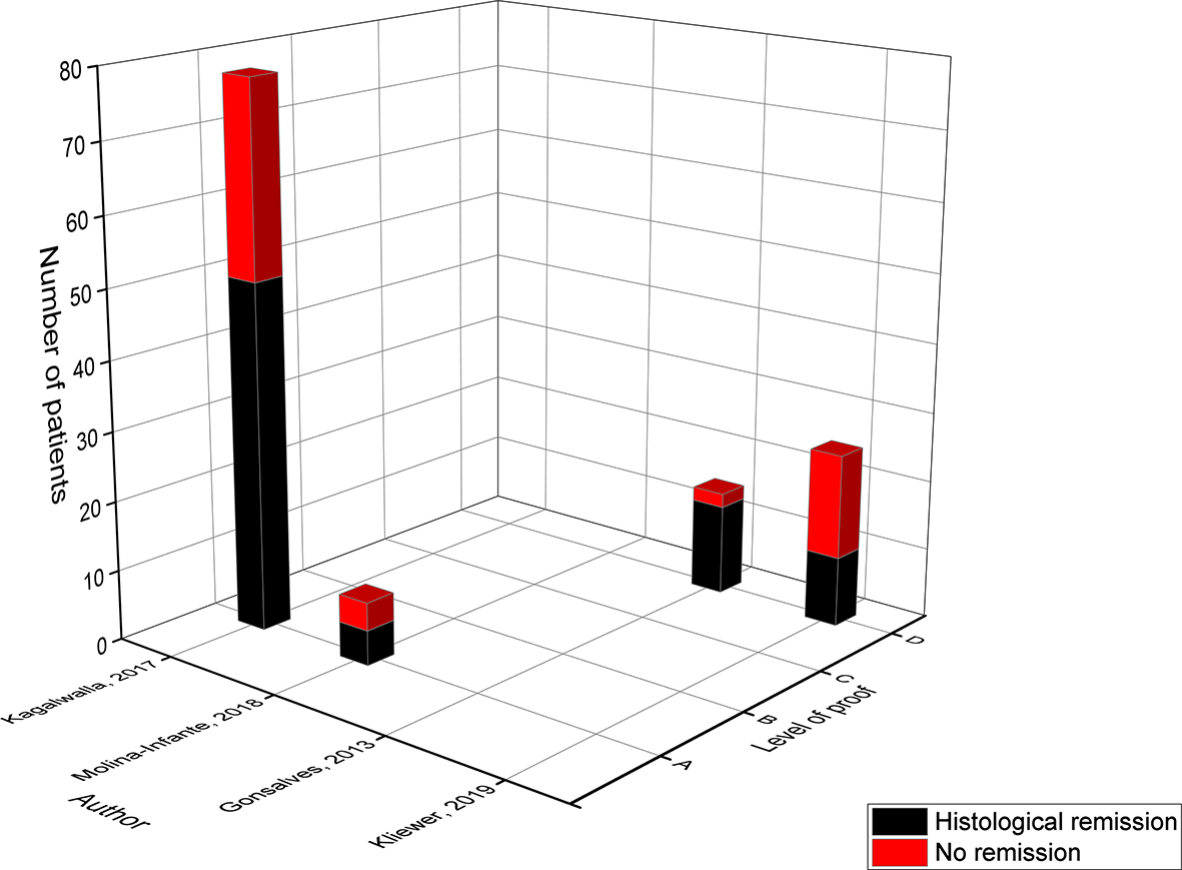
Level of proof of studies on the 4-food elimination diet.

The effectiveness of the 4-FED eliminating cow’s milk, wheat, egg and soy was evaluated in four prospective pediatric EoE studies, of which two were classified into level D as only an abstract was available. These studies included 127 children in total and achieved a mean histological remission of 62%, thereby approaching the 64% success rate of the 6-FED (**Table 5** and **Figure 5**) (17, 19, 25, 26).

**Table 5:** Overview of studies on the 4-food elimination diet.

Level	Author, publication year	Mean age (years)	n	Period	Duration of diet	PPI	Diet (Exclusion of)	Histological response (eos/HPF)		Remission rate
A	Kagalwalla, 2017	9,01 (1–18)	78	2011-2016	6-8 weeks	Yes, NR	Cow’s milk, wheat, egg & soy		< 15	64%
A	Molina-Infante, 2018	11 (5–13)	9	2014-2016	6 weeks	Yes, NR	Dairy products, gluten-containing cereals, egg, legumes & peanuts		< 15	57%
D	Gonsalves 2013	9	15	NA	6 weeks	NA	Cow’s milk, wheat, egg & soy		>50% reduction	87%
D	Kliewer, 2019	6-17	25	NA	12 weeks	NA	Cow’s milk, wheat, egg & soy		< 15	41%

NA, not applicable; PPI, proton pump inhibitors; NR, non-responder; R, responder; eos/HPF, eosinophils per high-power field.

The level A study of Kagalwalla in 2017 was the largest study with 78 participants following the 4-FED for 6-8 weeks and achieving a remission rate of 64% (19). In the study of Molina-Infante (level A) the 4-FED was part of the step-up dietary therapy as explained previously (17). Their modified 4-FED was stricter; eliminating all dairy products (milk, sheep & goat), gluten-containing cereals (wheat, barley, rye & oat), egg and legumes (peanuts, soy lentils, chickpeas, peas & beans), but reached only a success rate of 57% (17). This was attributed to a selection bias, since a part of the children who did not respond to the 2-FED already dropped out before they evolved to the 4-FED. The main effect in level D was observed within the study of Gonsalves in 2013 where 87% of the children underwent a reduction of more than 50% in eosinophil count (25). This effect was less prone when a cut-off level of ≤10 and ≤ 5 eos/HPF was applied achieving remission in respectively 60% and 47% of the children. The most recent study on the 4-FED by Kliewer in 2019 achieved the lowest remission rate (41%) in comparison to the three previously published studies (25). Despite the fact that only an abstract (NCT02778867) is available, this study is the first prospective multi-center randomized trial examining the effect of a diet on EoE. Since this is the golden standard to evaluate the efficacy of a new treatment, the results of this study could be of major importance but the reviewed full publication should be awaited. Of note, the duration of their dietary therapy was 12 weeks in comparison to 6 weeks in the other studies which could have affected the compliance to the diet.

#### The 4-FED Diet in Daily Practice

As was mentioned in the section of the 6-FED there is a variation in the treatment duration and interpretation of the 4-FED which complicates analyzing the effect of this diet in several studies. However, until now several trials have identified cow’s milk, egg, wheat and soy as the most common food triggers for pediatric EoE irrespective of the geographical area studied, thereby promoting the initial treatment with the 4-FED over the 6-FED (9, 12, 13, 16, 17, 19, 21, 23). In addition, both diets have variable remission rates ranging from 41% to 87% for the 4-FED and 52% to 81% for the 6-FED.

#### 2-Food Elimination Diet (2-FED)

**Figure 6:**
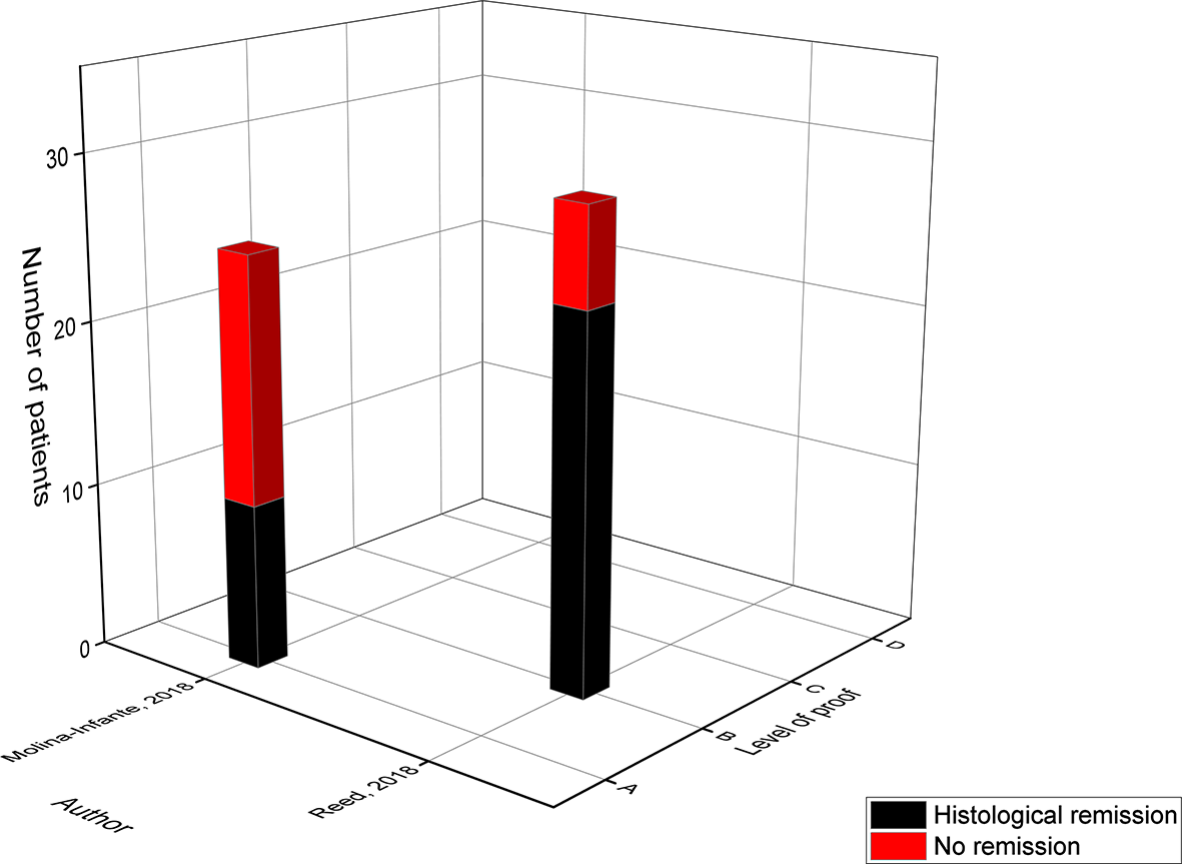
Level of proof of studies on the 2-food elimination diet.

Only two studies assessed the efficacy of the 2-FED (14, 17). The step-up study of Molina-Infante eliminated all dairy products and gluten-containing grains whereas the recent study of Reed only eliminated cow’s milk and soy while also studying the concomitant use of the 2-FED and tCS (**Table 6** and **Figure 6**) (14, 17). Since both studies interpreted the diet in a different way no general conclusion can be made on the effectiveness of eliminating only two food groups.

**Table 6:** Overview of studies on the 2-food elimination diet.

Level	Author, publication year	Mean age (years)	n	Period	Duration of diet	PPI	Diet (Exclusion of)	Histological response (eos/HPF)	Remission rate
A	Molina-Infante, 2018	11 (5–13)	25	2014-2016	6 weeks	Yes, NR	All dairy products & gluten-containing grains	< 15	40%
B	Reed, 2018	11,5 (1–18)	29	2014-2017	6 months (3 m tCS + 2-FED and 3m only 2-FED)	NR	Cow’s milk & soy (soy lecithin and soy oil were permitted)	< 15	79% (tCS + 2-FED 33% (2-FED only)

NA, not applicable; PPI, proton pump inhibitors; NR, non-responder; R, responder; eos/HPF, eosinophils per high-power field; tCS, topical corticosteroids; 2-FED, two-food elimination diet.

In 2018, the level B study of Reed assessed the combined treatment of tCS with a 2-FED for 3 months followed by an additional 3 months on the 2-FED alone (14). The concomitant use of tCS with a 2-FED resulted in a high remission rate of 79%, but when tCS were discontinued only 33% of the children maintained full histological remission. Of note, only 15 of the 29 children continued with the additional three months on the 2-FED alone. Regarding the level A study of Molina-Infante in 2018, only 40% achieved histological remission after eliminating all dairy products and gluten-containing cereals for six weeks (17).

In conclusion, no study was able to demonstrate the benefits of the 2-FED over the 4-FED. However, one may wonder whether different combinations of food most commonly associated with pediatric EoE (e.g. cow’s milk + wheat, egg + soy, wheat + soy…) in the 2-FED could have led to a higher effectiveness. In this respect it would be important to consider the food pattern in the geographical region studied, for example legumes appear to be an important culprit food for EoE in Spanish studies where they are regularly consumed, but less in American and Australian trials (17).

### Cow’s Milk Elimination Diet (CM-ED)

**Figure 7:**
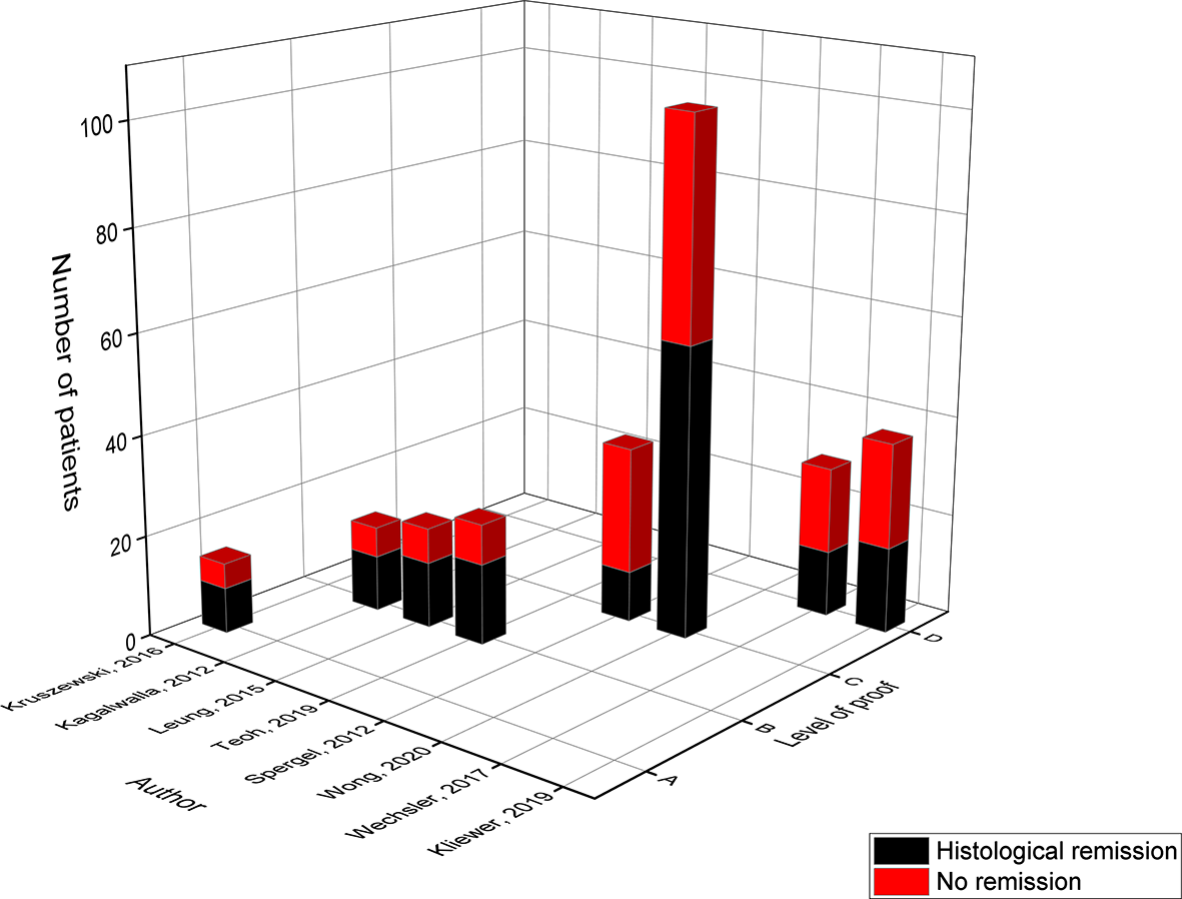
Level of proof of studies on the cow’s milk elimination diet.

Several studies have identified cow’s milk as the most prevalent food trigger for pediatric EoE (9, 11–13, 16, 17, 19, 21, 23). As a result, eight studies evaluated the single elimination of cow’s milk with a histological remission ranging from 30% to 67% (15, 16, 20–22, 24, 26, 28). In total, these studies included 291 children and obtained an average response rate of 53% (**Table 7** and **Figure 7**). Out of the eight studies evaluating the cow’s milk elimination diet (CM-ED) only four studies achieved a high remission rate comparable to that of the 4-FED (62%) and 6-FED (64%) (15, 16, 20, 22).

**Table 7:** Overview of studies on the cow’s milk elimination diet.

Level	Author, publication year	Mean age (years)	n	Period	Duration of diet	PPI	Histological response(eos/HPF)	Remission rate
A	Kruszewski, 2016	2-18	14	2012-2014	6-8 weeks	Yes	< 15	64%
B	Kagalwalla, 2012	5,5 (1–12)	17	2006-2011	6 weeks	R & NR	< 15	65%
B	Leung, 2015	13 (8–18)	20	2007-2013	8 weeks	Yes, NR	< 10	65%
B	Teoh, 2019	9 (1–16)	24	2013-2016	Median 3 months	NR	< 15	67%
C	Spergel, 2012	6,4 (1–18)	***35***	2000-2011	NA	NR	< 15	30%
C	Wong, 2020	9,9 (0,6-20,8)	102	NA	Mean 100,4 days	NA	< 15	57%
D	Wechsler, 2017	NA	30	NA	6-8 weeks	NA	< 15	43%
D	Kliewer, 2019	6 to 17	38	NA	12 weeks	NA	< 15	44%

NA, not applicable; PPI, proton pump inhibitors; NR, non-responder; R, responder; eos/HPF, eosinophils per high-power. Bolded value: the number of children in the intervention group was not specified, we therefore approached the unknown as stated in the method section “Analysis”.

In 2016, the level A study of Kruszewski compared the effect of the CM-ED to the intake of tCS and achieved histological remission in 64% of the children eliminating cow’s milk in comparison to 80% on tCS (22). A comparable success rate of 65% was achieved in the two level B studies of Kagalwalla in 2012 and Leung in 2015 (16, 22). In 2019, the level B study of Teoh evaluated the effect of a strict CM-ED in which all milk products including foods with traces were eliminated versus a liberalized CM-ED that allowed the ‘may contain’ and baked milk products (20). In general, only 58% of the children responded to both of the dietary therapies of which the highest success rate (67%) was achieved when following the strict CM-ED in comparison to only 29% of those on the liberalized CM-ED (20). Interestingly, eight of the 16 children who reached full remission on the strict CM-ED switched to a liberalized CM-ED whereby 63% remained in remission (20).

The level C study of Wong was the largest study where 102 children followed the CM-ED of which only 57% achieved remission (24). However, higher but not significant remission rates were achieved after 10 weeks of dietary therapy (81.8%) compared to 10-12 weeks (50%) and over 12 weeks (55.1%) of treatment (24). Again, children above 12 years of age reached higher remission rates of 67.5% compared to children below 6 years (59.3%) or between 6-12 years (42.9%) of age (24). More importantly, this study is the first to show that concomitant use of PPI’s in children following a CM-ED significantly improved the success rate of the CM-ED diet from 57% to 88% (24). In contrast, the lowest remission rate was obtained by the study of Spergel in 2012 (level C), of which only 30% responded to the CM-ED (21). Comparable response rates were obtained by the two prospective studies of Wechsler in 2017 (43%) and Kliewer in 2019 (44%), that were classified into evidence level D as only an abstract was available (26, 28).

#### The Cow’s Milk Elimination Diet in Daily Practice

Based on the average response rate of each dietary therapy we can conclude that the CM-ED has the lowest efficacy (53%) in treating pediatric EoE in comparison to the ELED (96%), 6-FED (64%), 4-FED (62%) and allergy-directed elimination diet (58%). However, the CM-ED has shown to induce remission in approximately half of the treated children by eliminating a single food. In contrast to the 4-FED and 6-FED where four to six types of foods have to be eliminated before achieving a remission rate of 60%, which is in comparison to the CM-ED only a difference of 10%. Can we state that the CM-ED is in fact more effective as an initial dietary intervention for the management of pediatric EoE taking also feasibility into account, and that the 4-FED and 6-FED should rather be opted as a rescue therapy for children who had no histological response? Moreover, a modeling-based analysis in pediatric and adult EoE has identified a step-up therapy starting with only eliminating dairy as the most effective approach to identify the culprit food item (34).

### The Sequential Reintroduction of Eliminated Foods

Of the 20 studies evaluating the different elimination diets for the treatment of pediatric EoE only seven studied the subsequent reintroduction of the eliminated foods by endoscopy (**Table 8**) (11, 13, 16, 17, 19, 21, 23). After reintroduction, all seven studies identified cow’s milk as the most common food trigger of EoE (11, 13, 16, 17, 19, 21, 23). In six of the seven studies cow’s milk was followed by egg, wheat and soy/legumes (12, 14, 18, 21, 22, 24). This differs from the study of TE Gómez who identified nuts as the second and fish/prawns as the third most prevalent culprit food (11). Of note, milk-induced EoE was present in 16 of the 22 children while only a small number of participants were found to have nuts (4/22) or fish/prawns (1/22) as their food trigger for EoE (11). They also included children with concomitant food allergies who were already avoiding eggs and/or legumes (11). The geographical area of these studies was focused in the United states, Spain and Italy (12, 14, 18, 21, 22, 24).

**Table 8:** Overview of studies evaluating the reintroduction of previously eliminated foods.

EvidenceLevel	Author, publication year	Patients (n) undergoing food reintroduction	Duration of each food reintroduction	Diet	Most common culprit foods identified by reintroduction

A	Kagalwalla, 2017	47/78	8 weeks	4-FED	Milk (85%) – egg (35%) – wheat (33%) – soy (19%)
A	Molina-Infante, 2018	64/73	6 weeks	2-FED 4-FED 6-FED	Milk (81%) – wheat/gluten (43%) – egg (15%) –legumes (9%)
A	TE Gómez, 2019	15/22	6 weeks	Directed	Milk (94%) – nuts (24%) –fish/prawns (6%)
B	Leung, 2015	22/81	8-12 weeks	ELED MFED	Milk – wheat – egg -beef
C	Henderson, 2012	51/98	Variable	ELED Directed 6-FED	Milk (65%) – egg (40%) – wheat (37%) – soy (38%)
C	Spergel, 2012	Not defined	Not defined	ELED Directed CM-ED 6-FED	Milk (35%) – egg (13%) – wheat (12%) – soy (9%)
C	Liacouras, 2005	Not defined	5 days	ELED Directed	Milk (45%) – egg (45%) – soy (38%) – corn (38%)

ELED, elemental diet; 2-FED, 2-food elimination diet; 4-FED, 4-food elimination diet; 6-FED, 6-food elimination diet; directed, allergy-test directed elimination diet; MFED, multiple food elimination diet.

Two of the seven studies evaluated the introduction of baked milk in children with milk-induced EoE, which has a lower allergenicity as heating destructs the conformational epitopes targeted by milk sIgE’s (16, 20). However, this hypothesis assumes that EoE is an IgE-mediated disease, which is still a matter of debate. In the study of Leung in 2015, 72% of the children remained in remission after introducing baked milk on a weekly basis while only 29% maintained remission in the more recent study of Teoh in 2019 (16, 20). Of note, in the study of Leung only children where dairy removal led to resolution of EoE and dairy reintroduction to recurrence followed the baked milk challenge (16). This differs from the study of Teoh who compared a strict elimination diet of cow’s milk for the first time versus a liberalized elimination diet which allowed baked goods (did not define frequency) but only eliminated the obvious sources of milk (20).

The time-period during which each food group was reintroduced was reported by four of the seven studies and ranged from 5 days to 12 weeks (11, 13, 17, 19). Hereby, only one study followed a fixed scheme for single food reintroduction starting with the introduction of soy followed by egg, wheat and finally cow’s milk (19). These food groups were sequentially introduced from least to most allergenic form for a time-period of 8 weeks (19). However, in the reintroduction phase individualization may be required (e.g. nutritional value, preference, culture…) which explains why some of the studies set the order of food reintroduction according to the preference of the parents and child.

Two studies questioned whether the outcome of a reintroduction could be different according to the product tested by an allergy test (21, 23). Hereby, they calculated the predictive values (PV) of the SPT and APT by assessing the single reintroduction of an eliminated food through endoscopy (21, 23). Concerning the four most common food triggers of pediatric EoE, cow’s milk had the highest positive PV (> 80%) to predict a successful reintroduction in contrast to egg, soy and wheat for which higher negative PVs were reported (egg 56%-90%, soy 64%-86% and wheat 67%-79%) (21, 23). The highest negative PV for each of these triggers was observed by the study of Spergel (21). In addition, this study evaluated the negative PV of the SPT, APT or combination of both skin tests to predict a successful reintroduction of cow’s milk, egg, wheat or soy but could not find a real difference (21). Finally, only five studies performed a SPT and/or Immunocap to assess a possible aeroallergen sensitization (9, 12, 16, 23, 27). In addition, Molina-infante and colleagues evaluated the implementation of a 2-FED during (n=51) and out (n=79) of the pollen season, but could not find a significant difference in histological remission rates (17). Of note, only 15 of the 130 patients had an oral allergy syndrome (OAS) to nuts and fruits of which all were already avoiding these foods before the start of the study (17).

## Discussion

Current analysis on the dietary therapies in the management of pediatric EoE reveals a lack of uniformity in the food groups that are being eliminated alongside with a variation in the treatment duration. In addition, six studies (10, 16, 17, 19, 22, 24) mentioned the concomitant use of PPI’s during dietary treatment, of which only two evaluated the effect on the histological remission (17, 24). Hereby, one study showed a significant 31% increase in the success rate of the diet (24). As a result, the remission rates of the dietary therapies in the remaining four studies could include the effects of PPIs which are known to have anti-inflammatory properties (10, 16, 19, 22). This lack of uniformity makes it difficult to draw general conclusions on the efficacy of these diets between trials. However, when solely considering the average response rate of these dietary therapies the ELED has the highest efficacy (96%) followed by the 6-FED (64%), 4-FED (62%), allergy-directed elimination diet (58%) and finally the CM-ED (53%).

Although the ELED is the most effective dietary treatment for pediatric EoE one has to consider the significant amount of disadvantages of this diet, including high costs, poor palatability, the need for a nasogastric tube, risk of a food allergy and negative impact on feeding and speech skills (7). Therefore, from the perspective of a clinician we would only prescribe this diet in formula fed infants or as a final rescue therapy in older children who did not respond to other dietary therapies. Concerning the three empiric elimination diets, it is remarkable that by solely eliminating cow’s milk 50% of the children experienced histological remission compared to 60% when four or six food groups were being eliminated. However, the composition of the 4-FED and 6-FED as well as the treatment duration differed between trials, which could have led to variable remission rates. Originally, the 6-FED consisted of cow’s milk, wheat, egg, soy, peanuts/tree nuts and fish/seafood (18). Though, several studies adjusted the composition of this diet and added the elimination of goat’s milk, sheep’s milk, corn, legumes and/or gluten or left out the elimination of fish and peanuts without evaluating the individual effect on the histological remission. After the elimination diet, the subsequent reintroduction of eliminated foods led to the identification of cow’s milk as the most common food trigger followed by either eggs, wheat and soy/legumes (12, 14, 18, 21, 22, 24). These findings support the step-up dietary strategy which builds up the amount of foods eliminated, rather than a step-down strategy where a highly restrictive diet is prescribed from the beginning.

Based on the results of the pediatric EoE trials that studied these three empiric elimination diets one could recommend clinicians to start with the CM-ED and gradually stepping up to the elimination of wheat, egg and soy/legumes in case of non-responders. In that order the elimination diet starts with high risk foods and gradually introduces lower risk foods to the dietary restrictions. In this respect, further research is necessary to examine the role of the cross-allergens of milk (goat’s milk & sheep’s milk) and wheat (gluten-containing cereals barley, rye & oat) as possible triggers for pediatric EoE. Until now, no research has been performed on the importance of these cross-allergens by eliminating them from the diet and evaluating the subsequent reintroduction by endoscopy. Secondly, the food group ‘legumes’ should be further defined as peanuts, lentils, chickpeas, peas, beans and lupins. However, only the study of Molina-infante described that these specific legumes had to be avoided while other studies used the general term (17). As a result, the added value of eliminating lentils, chickpeas, peas, beans and lupins from the diet is unknown. Finally, it would be important to consider the local food consumption pattern when prescribing the elimination diet since it has an influence on the allergy sensitization pattern. For instance, cow’s milk, wheat and eggs are part of the staple diet in Western countries in contrast to soy/legumes which are more often eaten in Mediterranean and Asian countries in addition to fish, seafood and treenuts. On the other hand, dairy products are less consumed in Asia due to the high prevalence of lactose intolerance (35). Overall, the geographical area of the incorporated pediatric EoE studies were focused in the United states, Spain and Italy. As a result, food triggers for pediatric EoE could differ in Asian countries where in addition EoE seems to be less prevalent (36).

For the allergy-directed elimination diet, studies showed a weak correlation between the allergy tests and the identification of the culprit foods triggering EoE. It should be noted that the results of the SPT and APT are influenced by skin test device and technique, threshold for considering a positive result, source of the allergen and the age of the child of which all varied substantially between the different trials (37). Secondly, the low predictive values of the SPT and APT could be due to the fact that the inflammatory sites are only found locally in the esophagus and not in the skin (38, 39). Furthermore, Immunocap and SPTs focus solely on identifying IgE-mediated reactions and the APT on non-IgE mediated reactions while there is still a debate on the possible role of IgE in the development of EoE. Ultimately, the evidence for the allergen-directed elimination diet as treatment for pediatric EoE seems to be too weak to be incorporated in daily clinical practice.

Despite the fact that studies have already shown seasonal exacerbations of EoE in children with comorbid allergic rhinitis (40), only the study of Molina-infante examined the effect of the pollen season on the efficacy of a diet and showed no negative influence on the remission rates (17). Importantly, only a handful of patients were diagnosed with OAS of which all were already avoiding the cross-reactive foods before enrollment. In addition, five studies performed SPT and/or Immunocap towards aeroallergens but none mentioned examining the role of aeroallergens in the pathogenesis of EoE (9, 12, 16, 23, 27). When seeing atopic children with EoE in clinical practice one might question whether pollen-food cross reactivity could be a possible trigger for EoE. In such a case performing the SPT and Immunocap could be a helpful tool to proof that aeroallergen sensitization could be related to an esophageal eosinophil accumulation. This is for instance important when discussing the possibility of consuming fruit during the ELED. Most of the studies that evaluated the ELED allowed the consumption of grapes and apples, which are known to cross-react with tree pollen. Finally, the clinician should consider that as for the atopic march aeroallergens seem to play a bigger role in EoE as the patients age increases while the role of food allergen sensitization decreases (41).

Until now, only a limited number of studies has evaluated and reported the sequential reintroduction of foods after an elimination diet when this in fact seems crucial for identifying the culprit foods triggering EoE. Furthermore, there is no consensus on the order and duration of the reintroduction phase which causes variability between studies. Recently, a protocol was suggested for the reintroduction of foods after following an ELED consisting of 6 predefined reintroduction groups (42). This protocol could be used in the future to guide clinical centra through the reintroduction phase after the ELED and empiric elimination diets. As stated in this report, the authors advise to start with boiled green vegetables, squashes and tubers, other non-legume vegetables and miscellaneous non-antigenic ingredients which were defined as group A1. Thereafter children can try to eat the listed vegetables of A1 raw before proceeding to group A2. In group A2, raw citrus and tropical fruits, melons berries and stone fruits are introduced. However, caution is advised in children with OAS due to a possible cross-reactivity with pollen-allergens. The next three groups may be initiated according to personal preference in which group B contains of lower risk starches and pseudo-grains, group C of poultry and group D of legumes, grains, seeds and animal proteins. Hereby, each predefined group (A, B, C or D) is introduced for 6-8 weeks before proceeding to the next phase. Clinical evaluation is advised 6-12 weeks after the last food is introduced within a predefined group, whereas endoscopy is suggested periodically and before proceeding to the last phase. The last group E consists of seven high-risk foods whereby a repeated endoscopy is advised between each food introduction starting with fish/shellfish, tree nuts, peanut, soy, egg, wheat and finally milk. It should be stated that serial endoscopies limit the practical implementation of this protocol. However, transnasal endoscopies may reduce the need for sedation in some pediatric patients and thereby limit the burden of repeated endoscopies (42).

We studied the role of dietary treatment in pediatric EoE based on a self-defined level of proof. Seven of the incorporated studies were prospective and hence classified as level A. Interestingly, only one prospective study analyzed the effect of the single CM-ED or the 6-FED. Respectively, nine studies were retrospective of which five classified into level B due to the intensive dietary counseling and four into level C since dietary follow-up data were lacking. Hereby, level C contained most of the studies with a high number of patients in contrast to level A and B which contained mostly the smaller studies. From four studies only an abstract was available so despite of their study design these were categorized as level D.

In conclusion, dietary counseling remains a challenge in pediatric EoE. Although the avoidance of cow’s milk appears to be effective for 50% of the children, the remaining half probably requires a more personalized dietary treatment plan. As limited positive predictive parameters exists to indicate the additional food products that need to be eliminated, clinicians should hope that future research creates clarity on this chronic debilitating condition.

## Author Contributions

LDV drafted the review, which was critically revised and edited by DMAB, IH and TV. LS, LN and DMAB substantially contributed to the conception and design of the review and interpreting the relevant literature. All authors revised the manuscript and approved the final version.

## Funding

DMAB and TV are a recipient of a senior researcher fellowship from the Fund for Scientific Research Flanders (FWO).

## Conflict of Interest

IH received funding from Nestlé, Nutricia and Takeda. TV has provided scientific advice and has served on the speaker bureau for Dr. Falk Pharma. DMAB received consulting fees from Avène, Thermo Fisher and Mead Johnson, and from Novartis and ALK for clinical study participation. None of the COI of these co-authors are related to the current review manuscript.
